# Susceptibility profiles of *Nocardia spp.* to antimicrobial and antituberculotic agents detected by a microplate Alamar Blue assay

**DOI:** 10.1038/srep43660

**Published:** 2017-03-02

**Authors:** Pan Zhao, Xiujuan Zhang, Pengcheng Du, Guilian Li, Luxi Li, Zhenjun Li

**Affiliations:** 1 State Key Laboratory for Infectious Disease Prevention and Control and National Institute for Communicable Disease Control and Prevention, Chinese Center for Disease Control and Prevention, Collaborative Innovation Center for Diagnosis and Treatment of Infectious Diseases, Beijing 102206, China; 2 Department of Endocrinology, Beijing Chaoyang Hospital, Capital Medical University, Beijing 100020, China; 3 Institute of Infectious Diseases, Beijing Ditan Hospital, Capital Medical University, Beijing Key Laboratory of Emerging infectious Diseases, Beijing 100015, China

## Abstract

*Nocardia* species are ubiquitous in natural environments and can cause nocardiosis. Trimethoprim-sulfamethoxazole has long been the monotherapy treatment of choice, but resistance to this treatment has recently emerged. In this study, we used microplate Alamar Blue assays to determine the antimicrobial susceptibility patterns of 65 standard *Nocardia* isolates, including 28 type strains and 20 clinical *Nocardia* isolates, to 32 antimicrobial agents, including 13 little studied drugs. Susceptibility to the most commonly used drug, trimethoprim-sulfamethoxazole, was observed in 98% of the isolates. Linezolid, meropenem, and amikacin were also highly effective, with 98%, 95%, and 90% susceptibility, respectively, among the isolates. The isolates showed a high percentage of resistance or nonsusceptibility to isoniazid, rifampicin, and ethambutol. For the remaining antimicrobials, resistance was species-specific among isolates and was observed in traditional drug pattern types. In addition, the antimicrobial susceptibility profiles of a variety of rarely encountered standard *Nocardia* species are reported, as are the results for rarely reported clinical antibiotics. We also provide a timely update of antimicrobial susceptibility patterns that includes three new drug pattern types. The data from this study provide information on antimicrobial activity against specific *Nocardia* species and yield important clues for the optimization of species-specific *Nocardia* therapies.

*Nocardia* species are ubiquitous in natural environments worldwide, including saprophytic components of fresh and saltwater, soil, dust, decaying vegetation, and animal excrement. *Nocardia* have been implicated in a variety of human infections and present in various clinical manifestations that are collectively termed nocardiosis, with symptoms ranging from localized skin and soft tissue infections to life-threatening pneumonia, central nervous system infections, and/or bacteremia^1^. Nocardiosis is a common opportunistic infection in immunocompromised patients that can be introduced through traumatic injury and usually presents as disseminated disease in AIDS patients^1^. Trimethoprim-sulfamethoxazole (SXT) has long been the monotherapy treatment of choice for nocardiosis^1^. However, two recent surveys of sulfonamide-resistant *Nocardia* spp. provided conflicting information; Brown-Elliott *et al*. found that only 2% of isolates were resistant to trimethoprim-sulfamethoxazole and/or sulfamethoxazole^2^, while Uhde *et al*. found that 61% were resistant to sulfamethoxazole and 42% were resistant to trimethoprim-sulfamethoxazole^3^. Furthermore, because the symptoms of nocardiosis are similar to those of tuberculosis (TB), misdiagnoses are common, and nocardiosis is often treated with antituberculotic antibiotics. It is therefore important to examine the susceptibility of *Nocardia* isolates to classic antituberculotic antibiotics and to evaluate the clinical outcome. In addition, both the diagnosis of nocardial pneumonia and the widely used SXT prophylaxis may result in resistance; thus, individualized treatment must be based on the results of *in vitro* drug susceptibility tests. However, data on antimicrobial susceptibility have lagged behind advances in taxonomy: species that are isolated less frequently in the clinical laboratory have not been systematically tested, and only a few reports provide data on newer antimicrobials^1^,^4^.

Knowledge of the general susceptibility pattern for a given pathogen is essential for the empirical treatment of infection, particularly when the results of laboratory tests are absent or delayed. Specific antimicrobial susceptibility patterns are predictable for several *Nocardia* spp., and they have been used to classify isolates into multiple distinct antibiotypes^1^. Routine antimicrobial susceptibility testing (AST) for *Nocardia* isolates includes the Etest and broth microdilution (BMD); in 2003^5^, the National Committee for Clinical Laboratory Standards (NCCLS) recommended BMD as the reference method. In 2010, Warren Lowman^6^ reported a comparative evaluation of BMD testing versus the Etest for several *Nocardia* species and other aerobic actinomycetes. They found that the Etest was not an acceptable alternative to BMD due to the dearth of data comparing the Etest to the reference method and the need for further epidemiological evaluation of aerobic actinomycetes. In 2014, McTaggart *et al*.^7^ reported the characterization of a variety of rarely encountered species by BMD and categorized them into four additional drug pattern types. However, determining minimal inhibitory concentrations (MICs) by the traditional BMD method is neither sufficiently rapid nor stable due to the expertise required.

In this study, we characterized the resistance of a variety of *Nocardia* isolates, including both standard and clinical strains. We determined the MICs of 32 antimicrobial agents, including both commonly used antimicrobial drugs and new clinical antimicrobials, against these *Nocardia* species and profiled their antimicrobial susceptibility patterns. Three new patterns were identified, providing highly valuable information for the clinical treatment of nocardiosis. Moreover, in this study we report the use of a broth-based method, the microplate Alamar Blue assay, for MIC determination of *Nocardia spp*. This assay was previously used for MIC determination for *Mycobacterium tuberculosis* and nontuberculous mycobacterial complex isolates with favourable results^8^,^9^. This method is faster, more stable, and more accurate than the traditional BMD or Etest methods^9^.

## Results

### Resistance observed in *Nocardia* isolates

Among the 32 antimicrobial agents (Table 1 and Table S1) of ten categories tested in this study, the isolates we tested showed high resistance to three categories: macrolides, clindamycin, and vancomycin (>70%). Resistance to tetracyclines and classic antituberculotic antibiotics was very common (>50%), and sensitivity to imipenem, meropenem, amikacin, linezolid, and SXT was very high (>85%) (Table S2).

In detail, 98% of isolates were susceptible to SXT and linezolid (standard strains ≥97%, clinical isolates 100%). Only the *N. wallacei* isolates were resistant to SXT (MIC = 64 mg/L). Further, 95% of our isolates were susceptible to meropenem (standard strains 95%, clinical isolates ≥93%), of which 60% of *N. otitidiscaviarum* and 75% of *N. brasiliensis* isolates were highly meropenem-susceptible, while all other isolates were sensitive or moderately susceptible. Further, 98% of isolates were susceptible to amikacin (standard strains 88%, clinical isolates 100%), whereas *N. amikacinitolerans, N. wallacei*, and *N. blacklockiae* isolates were highly resistant to amikacin (MIC ≥64 mg/L) (Tables S2 and S3).

In contrast, these *Nocardia* isolates showed low susceptibility to the antibiotic agents cefoxitin (18%), azithromycin (18%), tigecycline (19%), vancomycin (11%), rifampicin (6%), clindamycin (4%), ethambutol (4%), and isoniazid (0%).

### Sensitivity profiles by isolate source

A comparison between the two groups of clinical isolates and standard strains revealed different sensitivity profiles. In standard strains, the rates of sensitivity to ampicillin, cefepime, cefotaxime, ceftriaxone, and clofazimine were greater than 50%, and the sensitivity rates of ciprofloxacin and levofloxacin in clinical isolates were greater than 50%, revealing a distinguishing feature between these groups. All clinical *Nocardia* isolates were more susceptible to fluoroquinolones (ciprofloxacin, levofloxacin, ofloxacin, and moxifloxacin) than were the standard *Nocardia* isolates (Table S2).

By comparing MICs to 32 antimicrobial agents between the *N. farcinica* standard strains (14) and Chinese clinical isolates (11), the median MICs of ethambutol and cefmetazole for *N. farcinica* collected in China were higher than those for standard strains. Significant differences were observed for ethambutol (P = 0.012) and cefmetazole (P = 0.024). This result reveals that resistance among *N. farcinica* to antimicrobials is more severe in China.

### Comparison of antimicrobial resistance profiles between species

**Figure 1:**
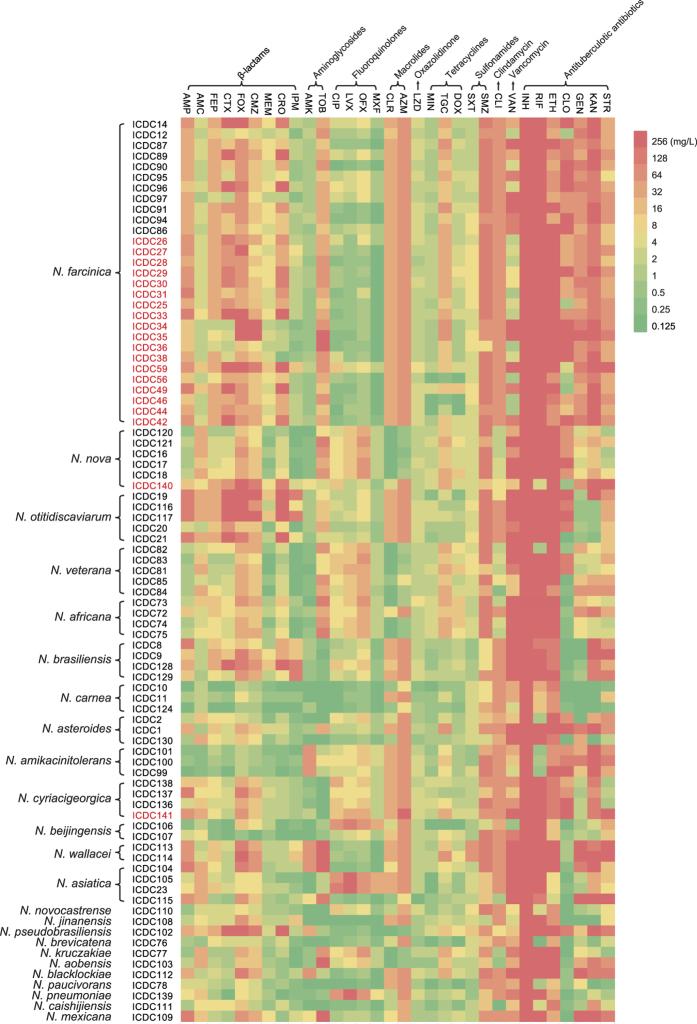
An MIC heatmap of 32 antimicrobial agents against 85 *Nocardia* isolates. The genera and isolate numbers are shown at left. The numbers in red are clinical isolates, and the others are standard strains. The abbreviations and categories of drugs are shown at top (AMP, ampicillin; AMC, amoxicillin-clavulanic acid; FEP, cefepime; CTX, cefotaxime; FOX, cefoxitin; CMZ, cefmetazole; MEM, meropenem; CRO, ceftriaxone; IPM, imipenem; AMK, amikacin; TOB, tobramycin; CIP, ciprofloxacin; LVX, levofloxacin; OFX, ofloxacin; MXF, moxifloxacin; CLR, clarithromycin; AZM, azithromycin; LZD, linezolid; MIN, minocycline; TGC, tigecycline; DOX, doxycycline; SXT, trimethoprim-sulfamethoxazole; SMZ, sulfamethoxazole; CLI, clindamycin; VAN, vancomycin; INH, isoniazid; RIF, rifampicin; ETH, ethambutol; CLO, clofazimine; GEN, gentamicin; KAN, kanamycin; STR, streptomycin).

**Figure 2:**
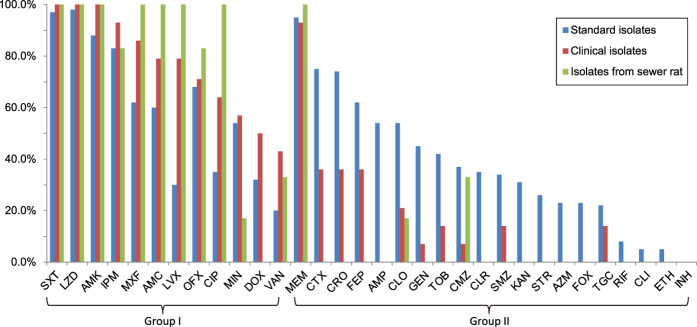
Differences in antibiotic susceptibility rates between clinical isolates, standard isolates, and those obtained from sewer rats. The 32 antimicrobial agents are grouped as follows: Group I, for which the susceptibility rate against clinical isolates was higher than that of standard isolates; Group II, for which the susceptibility rate against standard isolates was higher than that of clinical isolates. The drug abbreviations are the same as those in Fig. 1.

Among β-lactam antibiotics, the *Nocardia* strains showed high susceptibility to meropenem (95%) and imipenem (85%), which are classified as carbapenem antibiotics. All of the *N. farcinica, N. nova, N. veteran, N. africana, N. carnea, N. amikacinitolerans, N. cyriacigeorgica*, and *N. beijingensis* standard isolates were imipenem-susceptible, although these *Nocardia* strains showed various susceptibilities to other β-lactam antibiotics (Figs 1 and 2, Tables S2 and S3).

Among aminoglycoside antibiotics, high sensitivity to amikacin was observed, while only 34% of *Nocardia (N. carnea, N. brasiliensis, N. cyriacigeorgica, N. novocastrense*, and *N. jinanensis*) strains were susceptible to tobramycin.

*Nocardia* isolates showed high resistance to another older sulfonamide antibiotic, sulfamethoxazole, compared with SXT, whereas all of the *N. farcinica, N. otitidiscaviarum, N. africana*, and *N. wallacei* isolates were resistant to sulfamethoxazole.

Among tetracyclines, susceptibility to minocycline and doxycycline (52% and 33%, respectively) was higher than to the newer clinical antibiotic tigecycline (19%). However, none of the clinical sewer rat *Nocardia* isolates were susceptible to tigecycline or doxycycline. All of the *N. carnea, N. amikacinitolerans, N. asiatica, N. novocastrense, N. brevicatena, N. paucivorans*, and *N. caishijiensis* isolates were susceptible to minocycline and doxycycline (Fig. 1 and Table S3).

### Antimicrobial susceptibility patterns

In this study, we observed 10 antimicrobial susceptibility patterns that had been previously described by Brown-Elliott *et al*.^1^ and identified 3 new patterns. We did not obtain any clinical isolates of the *N. abscessus* complex (type I antimicrobial susceptibility pattern). *N. asiatica* isolates exhibited susceptibility similar to the type I antimicrobial susceptibility pattern that was designated type Ia; unlike classic type 1 isolates, these isolates were not susceptible to amoxicillin-clavulanic acid^7^. Notably, two *N. asiatica* isolates were resistant to moxifloxacin and had unusually high MICs (32 mg/L) (Table 2), similar to isolates reported by McTaggart *et al*. (8 mg/L)^7^. *N. otitidiscaviarum* and *N. brasiliensis* isolates also displayed distinct antimicrobial susceptibility patterns and were numbered types VII and VIII, respectively (Table 2). We also report antimicrobial susceptibility patterns for standard *Nocardia* species that were not categorized by the traditionally acknowledged groups and lack published MIC data (Table 2). These data and collective knowledge of the antimicrobial susceptibility patterns of these species are presented as preliminary findings to guide initial empirical therapies for nocardiosis.

In our analysis, *N. mexicana* and *N. pneumoniae* were grouped into a novel antimicrobial susceptibility pattern, type X, which is characterized by nonsusceptibility to amoxicillin-clavulanic acid and doxycycline. *N. amikacinitolerans* and *N. beijingensis* were grouped into a novel antimicrobial susceptibility pattern, type XI, which is characterized by nonsusceptibility to ciprofloxacin and clarithromycin. *N. carnea, N. novocastrense, N. jinanensis, N. blacklockiae*, and *N. caishijiensis* were grouped into a novel antimicrobial susceptibility pattern, type XII, which is characterized by susceptibility to many of the commonly used clinical antibiotics utilized in this study (Table 2).

### Resistance profiles to antituberculotic antibiotics

Because the symptoms of *Nocardia* infection are similar to those of tuberculosis^10^, which might result in misdiagnoses and erroneous treatment with antituberculotic antibiotics, we examined seven classic antituberculotic antibiotics, including rifampicin, isoniazid, streptomycin, ethambutol, gentamicin, clofazimine, and kanamycin, in this study. Among these antibiotics, we found that all *Nocardia* isolates were highly resistant to isoniazid (all MICs >256 mg/L) (Fig. 2 and Table S2). Only 6% and 4% of all *Nocardia* isolates were susceptible to rifampicin and ethambutol, respectively. All clinical *Nocardia* isolates were highly resistant to rifampicin and ethambutol, the MIC ranges of which were >256 mg/L and 64–256 mg/L, respectively (Fig. 2 and Table S2). Our standard *Nocardia* isolates showed various susceptibilities to other antituberculotic antibiotics (Table 2), but clinical *Nocardia* isolates were only susceptible to low levels of clofazimine and gentamicin, and they were resistant to kanamycin and streptomycin (Fig. 1).

## Discussion

This study focused on the antibiotic susceptibility patterns of different species and sources of *Nocardia* strains upon challenge with 32 antimicrobial agents. The data in this study provide detailed information on the antimicrobial activities of specific species of *Nocardia* isolates and yield important clues for the optimization of species-specific *Nocardia* anti-microbial therapies. We used a 96-well microplate Alamar Blue assay to test antibiotic susceptibility in this study. The MICs for control strains demonstrated a high degree of reproducibility, indicating that this assay is suitable for the routine determination of antimicrobial resistance patterns of *Nocardia* spp. in the clinical laboratory^9^. The technique described here can determine the MICs of antimicrobial agents within approximately 72 h. The microplate Alamar Blue assay is inexpensive and reliable for *in vitro* drug susceptibility testing of *Nocardia* isolates. Its application to *Nocardia* isolates could improve the international standardization of susceptibility testing methods.

Currently, SXT is the recommended first-line drug for the treatment of *Nocardia* infections^11^. The reported level of SXT resistance varies widely, ranging from 21% and 43%^2^,^12^,^13^ to >90%^6^,^7^,^14^,^15^,^16^. Larruskain *et al*.^12^ found that all *N. flavorosea* and approximately 50% of *N. carnea* and *N. farcinica* isolates exhibited SXT resistance. Similar to the results of a study by McTaggart *et al*.^7^, our results showed that 97% of *Nocardia* standard species were susceptible to SXT, and resistance was noted in two *N. wallacei* isolates (2/2). These discrepancies between studies could be attributed to geographic differences, the uncertain taxonomy of *Nocardia spp.* and species covered in different studies, the inherent growth characteristics of different species, the lack of a standardized testing method, and/or problems in determining the MIC, as has been noted by others^17^. Given the significant level of resistance that was noted in some studies, SXT susceptibility should continue to be monitored.

Although SXT is the drug most commonly used to treat *Nocardia* infections, its use is limited due to the fairly common occurrence of sulfonamide allergy. The main alternative is linezolid (oxazolidinone); others include amikacin (β-lactam), tetracycline, and ciprofloxacin (fluoroquinolone). In previous studies^3^,^6^,^12^,^13^,^14^,^15^, all *Nocardia* isolates were linezolid-susceptible, and almost all were amikacin-susceptible, except for some isolates of the *N. transvalensis* complex; most species were also imipenem-susceptible. In our study, linezolid, imipenem, and amikacin were effective against most *Nocardia* isolates. Larruskain *et al*. found that only 72% of *N. farcinica* isolates were imipenem-susceptible^12^, while all of the *N. farcinica* isolates in our study were imipenem-susceptible. For many other β-lactam antibiotics, resistance was species-specific (Fig. 1 and Table S3), as noted previously^3^,^12^,^13^,^16^,^18^. As in other studies, susceptibility to different members of the tetracycline family was uneven, and the overall sensitivity was low, while the rates of intermediate resistance were high^7^,^12^. A high proportion of resistance to glycopeptides, fluoroquinolones, macrocyclic lactones, and clindamycin has been noted by others^7^,^12^ and is further proved by the current findings. In the study by McTaggart *et al*.^7^, the susceptibilities of *N. farcinica* and *N. abscessus* isolates to ciprofloxacin were 50% and 100%, respectively, while none of the *N. nova* or *N. cyriacigeorgica* isolates were susceptible. Larruskain^12^ found that all of their *N. carnea* isolates were susceptible, while only 18% of *N. farcinica*, 2% of *N. nova*, and none of the *N. abscessus* and *N. cyriacigeorgica* isolates were susceptible. In our study, ciprofloxacin showed species-specific susceptibility: 55% of *N. farcinica* and none of the *N. nova* and *N. cyriacigeorgica* isolates were susceptible to ciprofloxacin. This result indicates that ciprofloxacin might remain an alternative when taxonomic identification is accurate or susceptibilities are known.

Knowledge of species-specific antimicrobial susceptibility patterns is important in assisting physicians with treatment options. As previously described^1^,^3^,^7^,^12^,^13^,^15^, we noted a strong coincidence between the drug pattern types described by Wallace *et al*.^17^ and McTaggart *et al*.^7^ The type Ia, II, III, IV, V, VI, and VIa drug patterns (Table 2) were displayed by *N. asiatica, N. brevicatena/N. paucivorans*, the *N. nova* complex, the *N. transvalensis* complex, *N. farcinica, N. asteroides*, and *N. cyriacigeorgica*, respectively. We also noted some discrepancies compared with previous studies. The *N. transvalensis* complex (type IV drug pattern) was imipenem-susceptible in Brown-Elliott’s study^1^ but not in the studies of Wallace and McTaggart, while the susceptibility was 50% in our study. Uhde^3^ reported a similar rate of resistance (52%) among their isolates. Brown-Elliott *et al*.^1^ and Wallace *et al*.^17^ found that *N. farcinica* was susceptible to imipenem and ciprofloxacin, while McTaggart *et al*.^7^ and others^3^,^8^,^12^,^15^ found that approximately half of the isolates were susceptible to these drugs. In our study, the susceptibilities of *N. farcinica* to these drugs were 88% and 60%, respectively. Brown-Elliott *et al*.^1^ found that *N. otitidiscaviarum* isolates (type VII drug pattern) were susceptible to ciprofloxacin, but most of the isolates in our study and those of Udhe *et al*.^3^ and McTaggart *et al*.^7^ were not susceptible. These results indicate that the susceptibility of the genus *Nocardia* is complicated, and more investigations are required to uncover the characteristics and mechanisms of antimicrobial resistance of this pathogen.

It is generally accepted that the incidence of nocardiosis is increasing, and the clinical symptoms are similar to those of tuberculosis, which could result in misdiagnoses^10^. Misdiagnosed patients are usually treated with antituberculotic antibiotics, but the therapeutic effects of these agents were unknown. We therefore analysed the susceptibility of *Nocardia* to seven types of classic antituberculotic antibiotics. Surprisingly, we found that most *Nocardia* strains, especially clinical strains, showed resistance to conventional antituberculotic agents. These results provide important guidance for clinical treatment and highlight the importance of fast and accurate diagnosis of *Nocardia* infections.

A limitation of our study was the low availability of less common clinical isolates for testing, which reduced the robustness of the antibiograms for some species. *Nocardia* is an opportunistic pathogen that can cause serious infections, especially in immunocompromised patients. In summary, our results show that SXT, meropenem, imipenem, linezolid, and amikacin are the most active antimicrobial agents against *Nocardia* strains, while most *Nocardia* isolates are highly resistant to isoniazid. Different drug patterns have been discovered in different species, yielding important clues for the optimization of species-specific *Nocardia* therapy. Thus, accurate taxonomic identification or susceptibility testing of clinical isolates should always be performed prior to treatment when possible. In addition, limited data have been reported to describe the genetic basis of antimicrobial resistance in the genus *Nocardia* (e.g., mutations in *gyrA* and *gyrB* encoding DNA gyrase and causing fluoroquinolone resistance as well as the carriage of genes encoding β-lactamases causing β-lactam resistance)^19^. Thus, detection procedures should be further evaluated to ensure their reliability, and more work is required to characterize the distribution and properties of antimicrobial resistance-associated genes and mutations in the genus *Nocardia*.

## Methods

### Strains and culture methods

In total, 85 *Nocardia* isolates were included in this study. Sixty-five standard *Nocardia* strains were obtained from the Leibniz Institute DSMZ-German Collection of Microorganisms and Cell Cultures (Braunschweig, Germany), 14 clinical isolates were isolated from 14 patients between 2010 and 2015, and 6 were isolated from the lungs of sewer rats in China in 2011. The *16 S rDNA* gene of all isolates was sequenced, and a nucleotide similarity of 97% with the reference sequences of each species was used for taxonomic identification by BLAST. Three strains of other genera, including *Staphylococcus aureus* ATCC 29213, *Pseudomonas aeruginosa* ATCC 27853, and *Escherichia coli* ATCC 35218, were used as controls^20^.

### Antimicrobial susceptibility test

Antibiotic susceptibility tests were performed using Alamar Blue assays on 96-well microplates to characterize the resistance profiles of these isolates to 32 antimicrobial agents (Table 1 and Table S1). Antimicrobial categories included β-lactamase, aminoglycoside, fluoroquinolone, macrolide, oxazolidinone, tetracycline, sulfonamide, clindamycin, vancomycin, and classic antituberculotic antibiotics. The Alamar Blue assay is an improved antibiotic susceptibility test based on BMD; Alamar blue is used as a colour-change indicator, and the step-by-step procedure and underlying mechanisms of action are shown in Text S1.

### Statistical analysis

We used the MICs for 50 and 90% of isolates (MIC50 and MIC90, respectively) and the MIC range to describe the sensitivity profile. MIC50 is defined as the MIC of a given agent that inhibits the growth of 50% of the isolates, while MIC90 is defined as the MIC of a given agent that inhibits the growth of 90% of the isolates. The MIC data were collected, stored, and analysed using SPSS 16 software. The ratios of the sensitivity profile and the difference of MICs in different groups (e.g., clinical isolates and standard strains) were compared. The distributions of MIC values have been tested previously and shown not to follow a normal distribution. The significance of differences between groups was therefore tested using the Mann-Whitney U test (P < 0.05).

## Additional Information

**How to cite this article**: Zhao, P. *et al*. Susceptibility profiles of *Nocardia spp*. to antimicrobial and antituberculotic agents detected by a microplate Alamar Blue assay. *Sci. Rep.* **7**, 43660; doi: 10.1038/srep43660 (2017).

**Publisher's note:** Springer Nature remains neutral with regard to jurisdictional claims in published maps and institutional affiliations.

## Supplementary Material

Supplementary Information

## Figures and Tables

**Table 1:** MIC breakpoints (mg/L) and concentration ranges of the 18 antimicrobials studied (an additional 14 antimicrobials are shown in Table S1 according to the CLSI interpretive criteria^20^).

Antimicrobial agents	MIC breakpoints			Concentration range	References
	Susceptible	Intermediate	Resistant		
Moxifloxacin	≤1	2	≥4	64–0.125	12
Trimethoprim-sulfamethoxazole^a^	≤32			256–0.125	20
Sulfamethoxazole^b^	≤32		≥64	128–0.125	20
Clindamycin	≤0.5	1–2	≥4	64–0.125	12
Tigecycline	≤1			64–0.125	12
Vancomycin	≤2	4–8	≥16	256–0.125	12
Kanamycin^b^	≤4			256–0.125	
Levofloxacin^b^	≤1			256–0.125	
Clofazimine^c^	≤1			256–0.125	21
Azithromycin^b^	≤2			64–0.125	
Ofloxacin^b^	≤1			64–0.125	
Rifampicin^c^	≤1			256–0.125	20
Isoniazid^c^	≤5			256–0.125	5
Streptomycin^b^	≤4			256–0.125	
Ethambutol^c^	≤5			256–0.125	22
Cefoxitin^b^	≤8			256–0.125	
Meropenem^b^	≤8			64–0.125	
Cefmetazole^b^	≤8			256–0.125	

^a^The susceptible breakpoint of trimethoprim-sulfamethoxazole is ≤2/38 mg/L according to the CLSI interpretive criteria^20^; however, the ratio of drug concentration we purchased was 6/26 mg/L when the mixed drug concentration was 32 mg/L. Thus, we set ≤32 mg/L as the susceptible breakpoint for trimethoprim-sulfamethoxazole. ^b^Breakpoints are approximations referring to published data for the same class of antibiotics, as there are currently no CLSI interpretive criteria. ^c^Breakpoints are approximations referring to published data for the breakpoints for *Mycobacterium tuberculosis*, as there are currently no CLSI interpretive criteria. ^*^The susceptible breakpoints and concentration range of an additional 14 antimicrobials, including amikacin, amoxicillin-clavulanic acid, ceftriaxone, ciprofloxacin, clarithromycin, imipenem, linezolid, minocyclin, tobramycin, cefepime, cefotaxime, doxycycline, ampicillin, and gentamicin, are shown in Table S1 according to the CLSI interpretive criteria^20^.

**Table 2:** Correlation between antimicrobial susceptibility profiles and *Nocardia* species designation.

Drug pattern type^a^	*Nocardia sp.*	No. of strains	Antimicrobial susceptibility profile^b^	
			Nonsusceptible (% intermediate or resistant)	Susceptible (%)
Ia^c^	*N. asiatica*	2	Ciprofloxacin, moxifloxacin, clarithromycin, amoxicillin-clavulanic acid, clindamycin, tigecycline, vancomycin, kanamycin, levofloxacin, azithromycin, ofloxacin, rifampicin, isoniazid, streptomycin, and ethambutol	Ceftriaxone, cefepime (50), tobramycin, amikacin, doxycycline, linezolid, imipenem, SXT, minocyclin, sulfamethoxazole, ampicillin, gentamicin, clofazimine, cefoxitin, meropenem, and cefmetazole
II^a^	*N. brevicatena, N. paucivorans*	2	Kanamycin MICs low (<1 μg/ml), clarithromycin clindamycin, vancomycin, ethambutol, rifampicin, isoniazid, and azithromycin	Ampicillin, amoxicillin-clavulanic acid, ceftriaxone, linezolid, amikacin, imipenem, ciprofloxacin, Minocyclin, moxifloxacin, SXT, tobramycin, cefepime, cefotaxime, doxycycline, tigecycline, kanamycin, levofloxacin, clofazimine, ofloxacin, streptomycin, and meropenem
III^a^	*N. nova* complex^d^	16	Amoxicillin-clavulanic acid (68), tobramycin, doxycycline, ciprofloxacin (94), moxifloxacin (94), tigecycline, vancomycin, levofloxacin, ofloxacin, rifampicin, isoniazid, ethambutol, and cefoxitin	Ceftriaxone (94), cefepime (88), imipenem, amikacin, clarithromycin (94), linezolid, SXT, cefotaxime, ampicillin (94), azithromycin (88), and meropenem
IV^a^	*N. transvalensis* complex^e^	4	Imipenem (50), tobramycin, amikacin, doxycycline, clarithromycin (75), ampicillin, gentamicin, clindamycin, vancomycin, kanamycin, azithromycin, rifampicin, isoniazid, streptomycin, cefoxitin, and cefmetazole	Ceftriaxone, ciprofloxacin, moxifloxacin, linezolid, SXT (50), cefotaxime, levofloxacin, clofazimine, ofloxacin, and meropenem
V^a^	*N. farcinica*	23	Ceftriaxone (72), cefepime (80), tobramycin (96), doxycycline (68), ampicillin, clarithromycin, sulfamethoxazole (91), ampicillin, gentamicin (96), clindamycin, tigecycline (91), kanamycin, azithromycin, rifampicin, isoniazid, streptomycin, ethambutol, and cefoxitin (96)	Amoxicillin-clavulanic acid (84), amikacin, moxifloxacin (88), linezolid, imipenem (88), SXT, and meropenem; variable susceptibility toward ciprofloxacin (60)
VI^a^	*N. asteroides*	3	Ampicillin, amoxicillin–clavulanic acid, clarithromycin, ciprofloxacin, cefepime, ampicillin, clindamycin, vancomycin, azithromycin, rifampicin, isoniazid, and ethambutol	Ceftriaxone, amikacin, linezolid, imipenem, minocyclin, moxifloxacin, SXT cefotaxime, gentamicin, and meropenem
VIa^c^	*N. cyriacigeorgica*	3	Amoxicillin–clavulanic acid (77), ciprofloxacin, moxifloxacin, clarithromycin, doxycycline (77), ampicillin (77), minocycline, moxifloxacin, clindamycin, vancomycin, kanamycin, levofloxacin, clofazimine, azithromycin, ofloxacin, rifampicin, isoniazid, ethambutol, and cefoxitin	Ceftriaxone, imipenem, tobramycin, amikacin, linezolid, SXT, tobramycin, cefepime, cefotaxime, gentamicin, streptomycin, and meropenem
VII^c^	*N. otitidiscaviarum*	5	Ceftriaxone, ampicillin (80), amoxicillin-clavulanic acid, and imipenem (often resistant to all β-lactam antibiotics), ciprofloxacin (80), clarithromycin, sulfamethoxazole, cefepime, cefotaxime, clindamycin, vancomycin, azithromycin, ofloxacin, rifampicin, isoniazid, streptomycin, ethambutol, cefoxitin, and cefmetazole	kanamycin, amikacin, doxycycline (80), moxifloxacin, linezolid, SXT, and kanamycin
VIII^c^	*N. brasiliensis*	4	Cefepime, imipenem, doxycycline, ciprofloxacin (75), clarithromycin, ampicillin, clindamycin, vancomycin, kanamycin, levofloxacin, azithromycin, ofloxacin, rifampicin, isoniazid, streptomycin, ethambutol, and cefoxitin	Amoxicillin-clavulanic acid, tobramycin, amikacin, linezolid, SXT, moxifloxacin, gentamicin, and clofazimine
Ix^a^	*N. pseudobrasiliensis*	1	Kanamycin, ampicillin, minocycline, doxycycline, cefepime, amikacin, amoxicillin-clavulanic acid, ceftriaxone, sulfamethoxazole, cefotaxime, clindamycin, tigecycline, vancomycin, kanamycin, clofazimine, isoniazid, and ethambutol	Ciprofloxacin, clarithromycin, tobramycin, linezolid, imipenem SXT, moxifloxacin, levofloxacin azithromycin, ofloxacin, rifampicin, streptomycin, cefoxitin, meropenem, and cefmetazole
X^f^	*N. mexicana, N. pneumoniae*	2	Amoxicillin-clavulanic acid, doxycycline, clindamycin, tigecycline, vancomycin levofloxacin, azithromycin ofloxacin, rifampicin, isoniazid, ethambutol and cefoxitin	Ceftriaxone, cefepime, imipenem, amikacin, linezolid, SXT, minocyclin, cefepime, cefotaxime, clofazimine, and meropenem
XI^f^	*N. amikacinitolerans, N. beijingensis*	5	Ciprofloxacin, clarithromycin, clindamycin, levofloxacin, azithromycin ofloxacin, isoniazid, streptomycin, and ethambutol	Amoxicillin-clavulanic acid Ceftriaxone, cefepime, imipenem linezolid, SXT, cefotaxime, ampicillin, and meropenem
XII^f^	*N. carnea, N. novocastrense, N. jinanensis, N. blacklockiae, N. caishijiensis*	7	Clindamycin (88), vancomycin, azithromycin, rifampicin (88), and isoniazid	Amoxicillin-clavulanic acid, ciprofloxacin (86), ceftriaxone, cefepime, imipenem (86), tobramycin, amikacin (86), doxycycline (71), clarithromycin (71), linezolid, SXT, moxifloxacin, cefotaxime, clofazimine, and meropenem

^a^Described by Wallace *et al*.^17^ and/or Brown-Elliott *et al*.^1^. ^b^If no value is indicated, the susceptible or nonsusceptible percentage is 100%. Amikacin, amoxicillin-clavulanic acid, ceftriaxone ciprofloxacin, clarithromycin, imipenem, linezolid, minocycline, moxifloxacin, trimethoprim-sulfamethoxazole, tobramycin, cefepime, cefotaxime, and doxycycline are drugs recommended by the CLSI^20^. Sulfamethoxazole, kanamycin, levofloxacin, clofazimine, azithromycin, ofloxacin, rifampicin, isoniazid, streptomycin, ethambutol, cefoxitin, meropenem, and cefmetazole are drugs tested for the first time or have rarely been used against *Nocardia* strains. ^c^Consistent with the drug pattern type described by McTaggart *et al*.^7^ and assigned a number in this study. ^d^The *N. nova* complex contains strains of *N. nova, N. africana, N. kruczakiae, N. veterana*, and *N. aobensis*. ^e^The *N. transvalensis* complex contains strains of *N. transvalensis* and *N. wallacei*. ^f^New drug pattern type described in this study.
