# Supplementary material for: Susceptibility profiles of Nocardia spp. to antimicrobial and antituberculotic agents detected by a microplate Alamar Blue assay
Source: Sci Rep. 2017 Mar 2;7:43660. doi: 10.1038/srep43660 (PMC5333629; doi:10.1038/srep43660)
Supplement: Supplementary Information [file srep43660-s1.pdf]

Susceptibility profiles of *Nocardia spp.* to antimicrobial and antituberculotic agents detected by a microplate Alamar Blue assay

Pan Zhao[1¶], Xiujuan Zhang [2¶], Pengcheng Du [3¶], Guilian Li[1], Luxi Li[1], and Zhenjun Li[1&]

1. State Key Laboratory for Infectious Disease Prevention and Control, and National Institute for Communicable Disease Control and Prevention, Chinese Center for Disease Control and Prevention; Collaborative Innovation Center for Diagnosis and Treatment of Infectious Diseases, Beijing 102206, China

2. Department of Endocrinology, Beijing Chaoyang Hospital, Capital Medical University, Beijing 100020, China

3. Institute of Infectious Diseases, Beijing Ditan Hospital, Capital Medical University, Beijing Key Laboratory of Emerging infectious Diseases, Beijing 100015, China

[¶]These authors contributed equally to this work.

[&]Corresponding author

Prof. Zhenjun Li,

National Institute for Communicable Disease Control and Prevention, Chinese Center for Disease Control and Prevention

P.O. BOX 5, Changping, Beijing 102206, China

Tel/Fax: 00861058900760

E-mail: zhenjun3886@163.com

S1 Table. MIC breakpoints (mg/L) and concentration range of 14 antimicrobials according to the CLSI interpretive criteria[10].

Antimicrobial agents	MIC breakpoints			Concentration range
	Susceptible	Intermediate	Resistant	
Amikacin	≤8		≥16	64–0.125
Amoxicillin-clavulanic acid	≤8/4	16/8	≥32/16	32/16–0.25/0.125
Ceftriaxone	≤8	16–32	≥64	256–0.25
Ciprofloxacin	≤1	2	≥4	256–0.25
Clarithromycin	≤2	4	≥8	32–0.25
Imipenem	≤4	8	≥16	128–0.25
Linezolid	≤8			128–0.125
Minocyclin	≤1	2–4	≥8	256–0.125
Tobramycin	≤4	8	≥16	256–0.25
Cefepime	≤8	16	≥32	32–0.125
Cefotaxime	≤8	16–32	≥64	256–0.125
Doxycycline	≤1	2–4	≥8	256–0.125
Ampicilin	≤8	16	≥32	128–0.125
Gentamicin	≤4	8	≥16	256–0.25

Table S2. MIC_{50}, MIC_{90}, MIC range (mg/L) and % of *Nocardia* species (no. of isolates) susceptible to 32 antimicrobial agents.

Antimicrobial(s)[a]		Standard isolates (65)				Clinical isolates (14)				Isolates from sewer rat (6)				overall rate of sensitive
		MIC_{50}	MIC_{90}	MIC range	%	MIC_{50}	MIC_{90}	MIC range	%	MIC_{50}	MIC_{90}	MIC range	%	%
β-lactam antibiotics	AMP	>4	<128	0.125–>128	54	<32	>64	16–>128	0	>32	>64	32–128	0	41
	AMC	>4/2	<32/16	<0.25/0.125–>32/16	60	<4/2	>8/4	2/1–32/16	79	>8/4	>4/2	4/2	100	65
	FEP	>4	<64	<0.125–>32	62	>16	>64	2–>32	36	>32	>32	>32	0	53
	CTX	<4	<128	<0.125–>256	75	<32	>256	2–>256	36	>32	<128	16–128	0	63
	FOX	>16	>128	0.5–>256	23	>64	>256	16–>256	0	>32	<128	32–256	0	18
	CMZ	>8	<64	<0.125–>256	37	<32	>128	8–>256	7	>8	<16	8–16	33	32
	MEM	<1	>4	<0.125–16	95	<4	<8	2–16	93	>4	<8	4–8	100	95
	CRO	>2	>64	<0.125–>256	74	<16	>256	0.25–>256	36	>32	>64	32–128	0	63
	IPM	>0.25	<16	<0.125–>128	83	<1	<4	0.25–16	93	>2	<4	2–8	83	85
aminoglycoside antibiotics	AMK	>0.25	<16	<0.125–>64	88	>0.5	<2	0.5–8	100	>4	<8	0.5–1	100	90
	TOB	<4	>256	<0.125–>256	42	>16	>256	<0.125–256	14	<32	<64	16–64	0	34
fluoroquinolones antibiotics	CIP	<2	>16	<0.125–128	35	>0.5	<8	<0.125–16	64	<0.5	<1	0.25–1	100	44
	LVX	>2	>32	<0.125–>256	30	>0.5	<4	0.25–32	79	>0.25	>0.5	0.5	100	43
	OFX	>4	<64	0.125–>64	68	>1	<8	0.5–32	71	>16	>32	0.5–4	83	70
	MXF	<0.5	<4	<0.125–32	62	<0.125	<1	<0.125–2	86	0.125	0.125	≤0.125	100	69
macrolide antibiotics	CLR	<16	>256	<0.125–>32	35	>256	>256	4–≥32	0	<64	<128	≥32	0	27
	AZM	>64	>256	0.25–>64	23	>64	>64	>64	0	>64	>64	>64	0	18
oxazolidinone antibiotic	LZD	>1	<4	0.125–8	98	0.5	<4	2–8	100	>1	<2	1–4	100	98
tetracycline antibiotics	MIN	<1	>2	<0.125–4	54	>0.5	<4	<0.125–4	57	>1	<2	1–2	17	52
	TGC	>4	>32	<0.125–64	22	>8	<128	0.25–64	14	>16	>32	16–32	0	19
	DOX	>1	<8	<0.125–16	32	<1	<8	<0.125–16	50	<4	>16	2–8	0	33
sulfonamide antibiotics	SXT	>2	<8	0.5–64	97	<4	<8	4–8	100	>4	<8	8	100	98
	SMZ	>64	>256	2–>128	34	<128	>256	8–>128	14	>128	>128	>128	0	28
clindamycin	CLI	<128	>256	<0.125–>64	5	>32	>64	8–64	0	>64	>64	>64	0	4
vancomycin	VAN	<128	>256	0.5–>256	2	<16	>256	1–>256	43	>2	<8	1–8	33	11
classic antituberculotic antibiotics	INH	>256	>256	>256	0	>256	>256	>256	0	>256	>256	>256	0	0
	RIF	>256	>256	<0.125–>256	8	>256	>256	>256	0	>256	>256	>256	0	6
	ETH	>128	>256	0.25–>256	5	<256	>256	64–>256	0	>256	>256	>256	0	4
	CLO	<2	>256	<0.125–>256	54	>32	>256	0.25–>256	21	>16	>128	1–256	17	46
	GEN	>4	>128	<0.125–>256	45	>32	>128	2–256	7	>32	>64	32–128	0	36
	KAN	<32	>256	<0.125–>256	31	<256	>256	128–>256	0	<128	<256	128–256	0	24
	STR	>8	<128	0.25–256	26	<64	<256	16–>256	0	<32	<64	16–64	0	20

[a] AMP, ampicillin; AMC, amoxicillin-clavulanic acid; FEP, cefepime; CTX, cefotaxime; FOX, cefoxitin; CMZ, cefmetazole; MEM, meropenem; CRO, ceftriaxone; IPM, imipenem; AMK, amikacin; TOB, tobramycin; CIP, ciprofloxacin; LVX, levofloxacin; OFX, ofloxacin; MXF, moxifloxacin; CLR, clarithromycin; AZM, azithromycin; LZD, linezolid; MIN, minocycline; TGC, tigecycline; DOX,

doxycycline; SXT, trimethoprim-sulfamethoxazole; SMZ, sulfamethoxazole; CLI, clindamycin; VAN, vancomycin; INH, isoniazid; RIF, rifampicin; ETH, ethambutol; CLO, clofazimine; GEN, gentamicin; KAN, kanamycin; STR, streptomycin.

Table S3-1. MIC range (mg/L) and % of different *Nocardia* species (no. of isolates ≥3) susceptible to 32 antimicrobial agents.

MIC/MIC range and % susceptible for species (type drug pattern) (no. of isolates≥3)										
Antimicr-obial(s)[a]	*N. farcinica* (V) (11)		*N. nova*(III) (5)		*N. otitidiscaviarum* (5)		*N. veterana* (5)		*N. africana* (4)	
	MIC range	%	MIC range	%	MIC range	%	MIC range	%	MIC range	%
AMK	0.5–2	100	0.125–0.25	100	0.5–1	100	0.125–0.25	100	0.125–1	100
AMC	1/0.5–4/2	100	16/8–32/16	0	32/16	0	4/2–32/16	40	4/2–16/8	75
CRO	2–256	30	1–8	100	>256	0	1–4	100	4–16	75
CIP	<0.125–8	55	4–8	0	1–4	20	4–16	0	4–8	0
CLR	16–>32	0	2	100	>32	0	<0.125–0.5	100	<0.125–8	75
IPM	0.25–2	100	<0.125–0.25	100	>128	0	≤0.125	100	<0.125–1	100
LZD	2–4	100	2–4	100	2–4	100	2	100	2–8	100
MIN	1–2	80	2–4	0	0.25–4	60	2–4	0	4	0
MXF	<0.125–1	100	2	0	0.5–1	100	1–2	20	2	0
SXT	2–8	100	4	100	4–8	100	4–8	100	2–4	100
SMZ	64–>128	0	32–>128	17	≥128	0	16–>128	40	≥128	0
TOB	16–64	0	8–128	0	4–32	20	8–64	0	32–128	0
FEP	4–>32	10	1–8	100	16–>32	0	1–8	100	8–16	50
CTX	2–>256	27	1–8	100	64–>256	0	1–8	100	4–8	100
DOX	1–4	9	4–16	0	0.25–2	80	2–16	0	8–16	0
AMP	16–64	0	0.5–2	100	4–>128	20	0.5–2	100	1–16	75
GEN	32–128	0	4–16	40	0.5–1	100	2–64	40	8–64	0
CLI	>64	0	2–4	0	8–>64	0	0.25–8	20	2–>64	0
TGC	16–64	0	16–32	0	1–4	60	16–32	0	16–32	0
VAN	2–>256	9	32–>256	0	128–>256	0	16–>256	0	>256	0
KAN	128–256	0	16–32	0	0.25–0.5	100	4–64	40	4–128	50
LVX	0.25–4	45	16	0	1–4	20	16–32	0	8–16	0
CLO	1–>256	9	64–256	0	<0.125–>256	20	<0.125–128	60	<0.125–0.5	100
AZM	>64	0	0.125–1	100	≥64	0	0.5–8	80	0.5–64	75
OFX	0.5–8	36	32–64	0	2–8	0	32–64	0	32–64	0
RIF	>256	0	>256	0	>256	0	>256	0	>256	0
INH	>256	0	>256	0	>256	0	>256	0	>256	0
STR	8–64	0	1–16	60	16–64	0	8–64	0	2–32	75
ETH	32–>256	0	≥256	0	≥256	0	64–>256	0	>256	0
FOX	8–256	9	16–64	0	16–>256	0	16–32	0	16–64	0
MEM	2–8	100	0.25–1	100	0.25–16	60	<0.125–0.5	100	0.25–2	100
CMZ	4–32	27	8–16	40	16–>256	0	8–32	20	16–32	0

Table S3-1. (continued)

N. brasiliensis (4)		*N. carnea* (3)		*N. asteroides* (VI) (3)		*N. amikacinitolerans* (3)		*N. cyriacigeorgica* (3)	
MIC range	%	MIC range	%	MIC range	%	MIC range	%	MIC range	%
0.5–1	100	<0.125–1	100	<0.125–1	100	≥64	0	0.25–0.5	100
1/0.5–2/1	100	2/1	100	>32/16	0	<0.25/0.125	100	8/4–16/8	33
32	25	<0.125–0.5	100	2–4	100	<0.125–0.25	100	2	100
1–2	25	0.125	100	8	0	4–8	0	8–16	0
≥32	0	0.5–32	67	32	0	>32	0	>32	0
16	0	≤0.125	100	0.25–0.5	100	0.25–0.5	100	1	100
2	100	0.25–1	100	1–2	100	4	100	4	100
0.5–1	75	<0.125–0.5	100	0.5–1	100	0.5–1	100	2–4	0
0.25	100	≤0.125	100	1	100	2–4	0	2	0
0.5–1	100	4	100	4	100	0.5–4	100	2	100
8–64	75	8–32	100	4–64	67	32–≥128	33	16–>128	33
<0.125-0.25	100	<0.125	100	<0.125–64	67	2–8	67	<0.125	100
8–>32	25	<0.125–1	100	16	0	0.5–2	100	4–8	100
4–>256	50	<0.125–0.5	100	4–8	100	<0.125–0.5	100	2–4	100
2–4	0	0.25–0.5	100	1–2	67	1	100	1–2	33
16–128	0	0.125–1	100	16–32	0	0.125–0.5	100	4–128	33
<0.125–1	100	<0.125	100	<0.125–128	100	16–125	0	1–2	100
≥64	0	>64	0	32–>64	0	>64	0	>64	0
1–2	50	0.5–4	67	1–64	33	8–16	0	1–8	33
≥256	0	4–32	0	16–>256	0	4–32	0	16–64	0
128–>256	0	<0.125	100	<0.125–>256	33	128–>256	0	64–>256	0
2–8	0	0.25–0.5	100	0.5–4	33	4–8	0	8	0
<0.125-0.25	100	<0.125–0.25	100	<0.125–>256	33	64–256	0	32–256	0
≥64	0	8–64	0	64	0	>64	0	>64	0
4–16	0	0.5	100	1–16	33	8–16	0	8–16	0
128–>256	0	16–32	0	>256	0	0.5–1	100	>256	0
>256	0	>256	0	>256	0	>256	0	>256	0
32–256	0	0.25–16	67	2–64	33	32–128	0	1–2	100
64–256	0	0.25–64	67	64–128	0	64–256	0	128	0
16–128	0	1–8	100	4–32	33	2	100	32–128	0
1–16	75	0.125–0.5	100	0.25–4	100	0.25–2	100	0.5–4	100
8–64	25	1–2	100	2–16	67	0.5–16	67	1–32	33

Table S3-2. MIC range (mg/L) of different *Nocardia* species (no. of isolates <3) to 32 antimicrobial agents*.

Antimicr-obial(s)	*N. beijingensis*	*N. wallacei*	*N. asiatica*	*N. transvalensis*	*N. novoca-strense*	*N. jinanensis*	*N. pseudobr-asiliensis*	*N. brevica-tena*	*N. krucza-kiae*
	(2)	(2)	(2)	(IV) (2)	(1)	(1)	(1)	(II) (1)	(1)
AMK	**<0.125**	>64	**<0.125**	**8**/64	**0.125**	**<0.125**	32	**<0.125**	**0.5**
AMC	**2/1/8/4**	**2/1/4/2**	≥32/16	≥32/16	**4/2**	**8/4**	16/8	**0.5/0.25**	32/16
CRO	**<0.125**	**2**	**0.25/2**	**0.5/2**	**1**	**2**	>256	**4**	**2**
CIP	2/64	**0.5/1**	64/128	**0.25/0.5**	16	**<0.125**	**<0.125**	**4**	8
CLR	4/16	≥32	≥32	**0.5**/8	**2**	>32	**<0.125**	4	**<0.125**
IPM	**0.125/0.25**	8/16	**0.25/0.5**	**2**	**0.5**	16	**2**	**0.25**	**<0.125**
LZD	**2**	**2**	**2**	**0.125/2**	**4**	**1**	**1**	**0.5**	**2**
MIN	**<0.125**/2	**1**/2	**<0.125**	**0.5**/2	**0.5**	2	4	**<0.125**	2
MXF	**1**/32	**0.125**	32	**<0.125**	**0.5**	**<0.125**	**<0.125**	**0.25**	2
SXT	**1**	64	**4**	**2**	**8**	**4**	**16**	**4**	**4**
SMZ	**4**/>128	>128	**16**	**16**/>128	>128	**2**	64	**16**	64
TOB	**0.5/1**	>256	**4**	128/256	**<0.125**	**<0.125**	**1**	**<0.125**	64
FEP	**0.25**	16	**4**/16	**1/8**	**4**	**4**	32	**8**	**1**
CTX	**<0.125**	**2/4**	**2/4**	**0.5/2**	**4**	**4**	>256	**4**	**1**
DOX	**<0.125**/4	2/8	**0.5**	2–4	**1**	2	8	**<0.125**	8
AMP	**0.25/4**	64	**4/8**	64/>128	**0.5**	**2**	32	**1**	**0.5**
GEN	**1/2**	≥256	**1/2**	128/256	**1**	**<0.125**	16	**2**	8
CLI	32/>64	>64	>64	1/>64	>64	>64	8	>64	1
TGC	**<0.125**/8	16/64	2/4	**1**/8	2	4	16	**0.5**	32
VAN	**0.5**/128	>256	>256	≥256	8	4	16	32	128
KAN	**1/4**	128/>256	32	≥256	**2**	**<0.125**	>256	**0.25**	**4**
LVX	4–128	**0.5/1**	≥256	**0.25**	4	**<0.125**	**0.25**	**0.5**	16
CLO	**<0.125/0.5**	**1**	**<0.125/0.25**	**<0.125/0.25**	**<0.125**	**<0.125**	128	**<0.125**	**<0.125**
AZM	≥64	64	64	8/>64	32	>64	**0.25**	64	**0.5**
OFX	8/>64	**1**	>64	**0.5**	8	**0.125**	**0.5**	**1**	64
RIF	>256	>256	>256	128/>256	>256	>256	**<0.125**	>256	>256
INH	>256	>256	>256	>256	>256	>256	>256	>256	>256
STR	16/32	256	8/16	16/256	8	128	**4**	**0.5**	64
ETH	16	>256	8	**4**/256	64	>256	32	>256	>256
FOX	**0.5**/16	**128/256**	**8**	64/128	64	>256	**4**	32	16
MEM	**0.5/2**	**2**	**1/2**	**1**	**2**	**4**	**1**	**0.5**	**1**
CMZ	**0.125/8**	32/64	**8**	16/64	16	128	**4**	16	16

Table S3-2. (continued)

N. aobensis	*N. blacklockiae*	*N. paucivorans*	*N. pneumoniae*	*N. caishijiensis*	*N. mexicana*
(1)	(1)	(II) (1)	(1)	(1)	(1)
**0.5**	>64	**<0.125**	**0.25**	**0.125**	**8**
32/16	**2/1**	**4/2**	16/8	**8/4**	16/8
**0.5**	**0.5**	**1**	**<0.125**	**4**	**0.5**
4	**0.25**	**<0.125**	64	**0.25**	**1**
**<0.125**	2	0.25	**0.25**	**2**	8
**<0.125**	**2**	**0.25**	**0.25**	**0.5**	**4**
**0.5**	**4**	**1**	**4**	**1**	**1**
**0.5**	2	**<0.125**	**1**	**0.25**	**1**
2	**<0.125**	**<0.125**	8	**<0.125**	**0.5**
**2**	**2**	**0.5**	**0.5**	**2**	**2**
**16**	>128	64	**16**	**16**	64
>256	**2**	**<0.125**	**0.5**	**<0.125**	>256
**2**	**4**	**2**	**1**	**8**	**2**
**1**	**2**	**1**	**<0.125**	**8**	**1**
2	4	**<0.125**	4	**0.25**	4
**2**	32	**4**	**8**	**1**	128
32	>256	8	**0.5**	**1**	>256
**0.5**	>64	>64	>64	**<0.125**	>64
16	32	**0.5**	2	**1**	2
32	>256	32	128	16	64
128	128	**<0.125**	**4**	64	>256
8	**0.5**	**<0.125**	256	**0.25**	2
**0.25**	**0.5**	**0.5**	**<0.125**	**0.25**	**<0.125**
**0.5**	>64	>64	64	32	32
16	**1**	**0.25**	>64	**0.5**	2
>256	**1**	>256	4	256	>256
>256	>256	>256	>256	>256	>256
128	**0.25**	**1**	**4**	128	16
>256	16	32	128	32	>256
128	**8**	**8**	16	64	64
**2**	**0.5**	**1**	**0.5**	**8**	**8**
32	**4**	**4**	**2**	32	32

*bold typeface indicates that the species was susceptible to the antimicrobial drug.

Text S1. Step-by-step procedure of Antimicrobial susceptibility test

Susceptibility testing was performed using the Mueller–Hinton II broth microdilution method[8, 9]. All tests for each strain were conducted twice at least. The isolates were grown on microplates. The inocula were prepared from actively growing bacteria collected from brain-heart infusion agar. The strains were then adjusted with saline to a bacterial cell density of 1.5×10^8 CFU/mL (0.5 McFarland standard), and then diluted 1:200 with Mueller–Hinton II Supplement (MH II-S) (MH-II broth + 5% ADC). Antibiotics were serially diluted two fold in 100 μL of MH-II-S. The range of antibiotic concentrations was 256–0.125 mg/L, except for: imipenem, linezolid, sulfamethoxazole, and ampicillin (128–0.125 mg/L); amikacin, moxifloxacin, clindamycin, tigecycline, azithromycin, ofloxacin, and meropenem (64–0.125 mg/L); amoxicillin-clavulanic acid (32/16–0.25/0.125 mg/L); and clarithromycin and cefepime (32–0.125 mg/L). The final volume in each well was 200 ul (100 μL of bacterial suspension and 100 μL of antibiotic solution). The overall performance of the susceptibility test system was monitored by testing the three quality control bacterial strains in each analysis.

A drug-free control well (MH II-S + inoculum) was used to determine when to add the Alamar Blue. A medium (MH II-S) without inoculum control well was used to measure the interference of MH II-S with the Alamar Blue. We also used a series of control wells in which all of the antibiotics, along their respective concentration gradients, were mixed with MH II-S to determine the degree of interference with the color of the Alamar Blue. The plates were sealed in individual Ziploc bags and

incubated at 37 ℃.

The indicator consisted of 20 μL of Alamar Blue and 50 μL of sterile 5% Tween-80. After 24 h, we used the indicator to examine the first drug-free growth control well and then re-incubated the plates for 8–12 h. Alamar Blue is a redox indicator that is used to evaluate metabolic function and bacterial health. A color change from blue to pink indicates bacterial growth. If the control turned pink completely, all of the other wells received the indicator. We recorded the colors of all the wells after a further 24 h of incubation. If the first drug-free growth control well did not turn pink, we used the indicator to examine the second drug-free control well and then repeated the above steps. The MIC was defined as the lowest concentration of a drug capable of inhibiting the visible growth of the tested isolates. We recorded the MIC as the lowest concentration of the drug that showed no color change. Each MIC was read on the 3rd or 2nd day. The MIC results for each drug for each isolate are the mean value from two tests. The MIC breakpoints of the drugs, indicating sensitivity, moderate susceptibility, and resistance, were interpreted according to the approved guidelines established by the National Committee for Clinical Laboratory Standards[10], except for: clofazimine (Table 1), where the approximations refer to the published breakpoints for *Mycobacterium tuberculosis*[20]; moxifloxacin, clindamycin, tigecycline, and vancomycin, which were assessed according to the method of Larruskain *et al.*[13]; and kanamycin (Table 1), which was assessed according to the approximations for the same class of antibiotics, as there are currently no CLSI interpretive criteria (Table 1).
